# *In medio stat virtus*: unanticipated consequences of telomere dysequilibrium

**DOI:** 10.1098/rstb.2016.0444

**Published:** 2018-01-15

**Authors:** Lea Harrington, Fabio Pucci

**Affiliations:** Wellcome Trust Centre for Cell Biology, School of Biological Sciences, College of Science and Engineering, University of Edinburgh, Mayfield Road, Edinburgh EH9 3JR, UK

**Keywords:** telomere dysequilibrium, histones, DNA methylation, cell senescence, cell differentiation

## Abstract

The integrity of chromosome ends, or telomeres, depends on myriad processes that must balance the need to compact and protect the telomeric, G-rich DNA from detection as a double-stranded DNA break, and yet still permit access to enzymes that process, replicate and maintain a sufficient reserve of telomeric DNA. When unable to maintain this equilibrium, erosion of telomeres leads to perturbations at or near the telomeres themselves, including loss of binding by the telomere protective complex, shelterin, and alterations in transcription and post-translational modifications of histones. Although the catastrophic consequences of full telomere de-protection are well described, recent evidence points to other, less obvious perturbations that arise when telomere length equilibrium is altered. For example, critically short telomeres also perturb DNA methylation and histone post-translational modifications at distal sites throughout the genome. In murine stem cells for example, this dysregulated chromatin leads to inappropriate suppression of pluripotency regulator factors such as *Nanog*. This review summarizes these recent findings, with an emphasis on how these genome-wide, telomere-induced perturbations can have profound consequences on cell function and fate.

This article is part of the theme issue ‘Understanding diversity in telomere dynamics’.

## 1. Background

Cellular senescence, or the cessation of cell division and entry into a quiescent but viable state, is an almost universally conserved stress response of eukaryotic cells. It can be induced in a number of ways that include by genome-wide DNA damage, critically eroded telomeres and activation of certain oncogenes [1]. For example, in cells lacking a means to maintain telomeres, the gradual erosion of this repetitive, G-rich DNA leads ultimately to loss of chromosome end-protection. This ‘uncapping’ of telomeres leaves telomeres vulnerable to degradation, recombination or end-to-end fusions, and leads prevalently, but not exclusively, to senescence in normal cells, and to cell death in cancer cells that have subverted certain DNA damage surveillance mechanisms [2,3]. To avoid cellular senescence, many cell types express an RNA-dependent reverse transcriptase that replenishes telomeric DNA, called telomerase reverse transcriptase (TERT). A wealth of evidence in both unicellular and multicellular organisms has established that telomerase activity acts to replenish telomeres [4]. In mammals, the ability to maintain or even elongate telomeres is developmentally regulated, and peaks during early development via both telomerase-dependent and telomerase-independent elongation mechanisms [5]. Later in development, transcriptional repression of the *TERT* gene occurs, and even in cell types and tissues that retain some telomerase activity, telomere erosion is evident with increasing age. In many genetic contexts, telomerase is haploinsufficient; one functional copy of the telomerase RNA or TERT is insufficient to prevent telomere erosion, uncapping or genome instability [6].

Among the findings to establish a first link between telomere erosion and cellular senescence were studies in the baker's yeast *Saccharomyces cerevisiae* and in human primary fibroblasts. Many unicellular organisms express telomerase constitutively; for example, *S. cerevisiae* populations are normally immortal and do not exhibit telomere erosion. However, a genetic screen for mutations leading to ‘ever shorter telomeres' established that telomere erosion leads to cellular senescence [7] and led to the identification of genes encoding key telomerase and regulatory protein subunits [8,9]. In human primary cells, telomere erosion was shown to correlate with the ‘mitotic clock’ that limits the number of times that cell populations double—thus providing a potential molecular basis for the lifespan limit that was first described by Leonard Hayflick in the 1960s [10–12]. Cellular lifespan can be extended for a time via inhibition of certain DNA damage checkpoints, but telomere erosion irrevocably leads to genome instability and apoptosis [13], except in rare clones that reacquire telomerase activity and become immortal [14]. Indeed, ectopic over-expression of *TERT* is sufficient to lengthen telomeres and immortalize many primary cell types [4]. Although re-expression of *TERT* is the most common mechanism for cells to overcome senescence, it is not the only means of doing so. A subset of cancers resolves the telomere-shortening problem by homologous recombination (HR)-based, alternative lengthening of telomeres (ALT) [15]. The molecular mechanisms underlying ALT are still not completely understood, however recent studies indicate that protein complexes such as NuRD-ZNF827 serve as a scaffold for homology-directed recombination [16]. In addition, recruitment of PCNA and RFC-5 to a double-strand break within telomeric DNA can promote telomere elongation via the recruitment of POL*δ* and the synthesis of new telomere DNA from an existing telomeric DNA template [17].

## 2. Is there a Goldilocks zone for telomere length?

Is there an optimal telomere length for cell function? This is a complex question because the factors that influence telomere length equilibrium are myriad [4,6], and can vary at the level of individual telomeres, cells, tissues or organisms. Equilibria are also dynamic, and may change with time, nutrient status, stress, environmental or intrinsic cues, or with cell state (see also [18,19]). There are known opposing forces that cooperate or compete to maintain average telomere length within an equilibrium range. Factors that erode telomeres include, but by no means are limited to, the end replication problem, enzymatic processing, oxidative stress, exogenous factors, genetic modifiers, telomere trimming, telomere looping, HR-driven telomeric rearrangements and impaired recruitment or the dosage of telomerase itself [20,21]. As discussed above, critically eroded telomeres can lead to a DNA damage response (DDR), with concomitant impacts upon cell division or survival. In some cases, these perturbations induce other catastrophic events such as chromosome loss, fusion or other rearrangements. These cellular aberrations may lead to organism-wide disease that includes infertility, cancer, early mortality and genetic anticipation (an increased risk of disease penetrance in following generations; [22], see also [23,24]). Indeed, short telomeres are not just associated with adverse healthspan in humans and other mammals, but emerging evidence also suggests relationships to brood size, egg health, survival and early mortality in other organisms such as birds and lizards [25–30].

The study of telomerase-deficient murine models *in vivo* [31,32] has underscored that it is critically short telomeres, not average telomere length *per se*, that affects genome stability. However, it is not just critically eroded telomeres that may harbour defects in telomere integrity; long telomeres can also be problematic. Factors that are known to drive telomere elongation are both telomerase-dependent and telomerase-independent (i.e. recombination-dependent) [4,6,33,34]. Telomere elongation can also be driven by environmental factors [35]. Replication fork progression (and fork stalling) through telomeres may affect both the length and integrity of chromosome ends [36–38]. Excessively long telomeres may also affect the fraction of telomeric DNA bound by a six-subunit telomere complex called shelterin [39]. Another complex required for telomere maintenance and telomerase recruitment, called CST (CTC1–STN1–TEN1), may become especially important at longer telomeres [40,41]. There are numerous phenotypes associated with excessively long telomeres in various organisms; for example, in ALT or telomerase-positive cancer cells, long telomeres lead to an increase in sensitivity to DNA damage [42,43] and to increased telomere fragility [44,45]. Indeed, the DDR itself is critical to telomere length homeostasis, and ataxia-telangiectasia mutant (ATM) activation stimulates telomere elongation [46–48]. Telomere elongation may also be a driving force in cancer progression. An increasing number of human cancer types exhibit mutations in the *TERT* promoter that lead to upregulated telomerase activity or mutations within shelterin components such as POT1, both of which often result in a longer average telomere length [49,50]. Thus, deviations from the norm (too long or too short) may both signal a loss of telomere integrity, although presumably through distinct mechanisms (as below; see also [51]).

As important as telomerase is to counter-balance telomere erosion, there exist equally important physiological mechanisms to counter-balance excessive telomere lengthening. Telomere recombination in yeasts, ciliates, plants and other organisms can lead to both rapid telomere deletion and expansion [52–55]. Mammalian telomeres are also subjected to active trimming mechanisms that generate excised, circular telomeric ssDNA called t-circles or c-circles (depending from which telomeric strand they are comprised) [45,56–58]. These circular intermediates also appear to be a characteristic feature of yeast strains lacking telomerase function [59–61]. In telomerase-positive cell types such as murine tissues and human embryonic stem cells (ESCs), telomere trimming is an important mechanism by which cells can fine-tune their telomere length [45,57,62]. Mechanisms that mediate telomere trimming in humans are likely to involve several factors, including Nbs1 and Xrcc3 [57,62–66], and the recently identified telomere-associated protein ZBTB48, or TZAP [67,68]. Thus, it appears that telomeres are under a constant flux of both lengthening and shortening, a process referred to as ‘telomere homeostasis' [4,6].

These and other data show that perturbations in telomere equilibrium, either to shorter or longer telomeres, impact several human diseases [20]. It has been proposed that telomere equilibrium must exist within an equilibrium zone that balances the deleterious effects of telomeres that are too long or too short [42]. By analogy to an astrophysics term for planets that might support life because they are neither too far nor too close to a sun, there may be a context-dependent, optimal telomere equilibria; a ‘Goldilocks zone’. This is an important question in the telomere field which has not yet been fully addressed. One recent study addressed this question in *S. cerevisiae*, using strains that possessed up to fivefold longer telomeres than wild-type strains. There was no measurable difference in lifespan or fitness under a variety of conditions [69,70]. In summary, much research remains to be done rigorously assess whether there is a ‘Goldilocks zone’ for telomere function.

## 3. Similar epigenetic alterations may occur in senescence, cancer and cell fate

The changes that accompany cell senescence and cancer progression are not limited only to telomeres. Other genome-wide alterations occur that exert a profound effect on gene expression and cell differentiation. As elaborated below, recent findings show that epigenetic alterations that affect the genome-wide expression patterns that contribute to cell senescence and cell fate have more in common with telomeres than previously appreciated.

One of the most critical ‘marks’ that affects gene expression in many organisms is DNA methylation. Mammalian gene promoters are enriched in the dinucleotide CpG, and methylation of this sequence is an important and dynamic regulator of gene expression and differentiation status. CpG methylation promotes the recruitment of histone methyltransferases and, at many genetic loci, these two epigenetic alterations establish a repressive state that stably inhibits gene expression. Conversely, removal or exclusion of DNA methylation at a promoter can result in chromatin remodelling and a permissive state for gene expression. Thirty years ago, an association between cancer, aging and DNA hypomethylation was first described [71–73]. Abundant evidence has since been accrued that genome-wide DNA hypomethylation, often accompanied by regional hypermethylation, is a shared phenotype of senescent and cancer cells that is evolutionarily conserved from mouse to human [74]. The methylomes in aged human tissues and cancer have been profiled molecularly, revealing conserved methylome ‘footprints’ that are potential biomarkers of ageing and cancer [75–77]. Such age-associated signatures in the DNA methylome have also been recently described in dogs and wolves [78]. These findings have led to the notion that there exists an ‘epigenetic’ or ‘methylome’ clock, and that this dynamic, age-associated change in genome-wide DNA methylation patterns could influence the ageing process itself and in turn be influenced by therapeutic interventions [79–81]. While many tissues do indeed show evidence of such age-associated alterations, some tissues, such as the murine hippocampus, do not exhibit any detectable change in DNA methylation or the expression of cytosine-modifying enzymes during ageing of either sex [82]. While the associations are tantalizing, the causal relationship between the methylome and ageing is still unclear and under active investigation.

Epigenetic alterations of the genome may also be as important to cancer development as they are to normal tissue development, and can arise via alterations in DNA methylation and chromatin organization [83]. These genome-wide changes drive transitions in cell lineage commitment that include, for example, trans-differentiation [84] or the epithelial-to-mesenchymal transition [85]. Cancer stem cells, which comprise rare cells that emerge during cancer development, also exhibit plasticity and heterogeneity in cell fate [86]. Thus, changes to the DNA methylation status of the genome (colloquially referred to as the ‘methylome’) can be viewed as a cellular history that distinguishes an early precursor cell fate from the fate of that cell type several divisions later. In summary, a dynamic methylome is integral to normal development, cancer development, cell differentiation and cell senescence.

**Figure 1.**
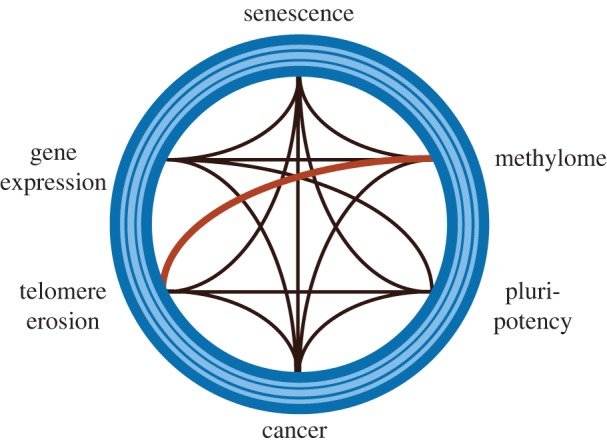
Schematic of the interconnectivity among telomeres, methylome maintenance and other fundamental cellular functions. A summary of known, interconnected cellular processes such as telomere shortening, pluripotency, cancer development, DNA methylation and changes in gene expression (black lines). However, as discussed in this review, recent findings uncovered a deeper and more direct relationship between telomere length maintenance and the methylome (red line).

Until recently, it was thought that the genomic changes that result in alteration of DNA methylation during senescence represented a mechanism that was distinct from telomere attrition. For example, although telomere erosion was known to induce alterations in telomeric and sub-telomeric DNA heterochromatin, such as loss of histone repressive marks and subtelomeric DNA hypomethylation, these effects were considered constrained to sequences proximal to the telomere [87–89]. Short telomeres were also known to impair the establishment of a differentiated state, for example, in neuronal stem cells, osteoblasts and induced pluripotent stem cells (iPS) [90–92]. However, recent studies in murine and human cell models suggest that the link between eroded telomeres and the methylome is more wide-reaching than appreciated previously (figure 1).

## 4. A direct link between short telomeres, DNA methylation and stem cell pluripotency

**Figure 2.**
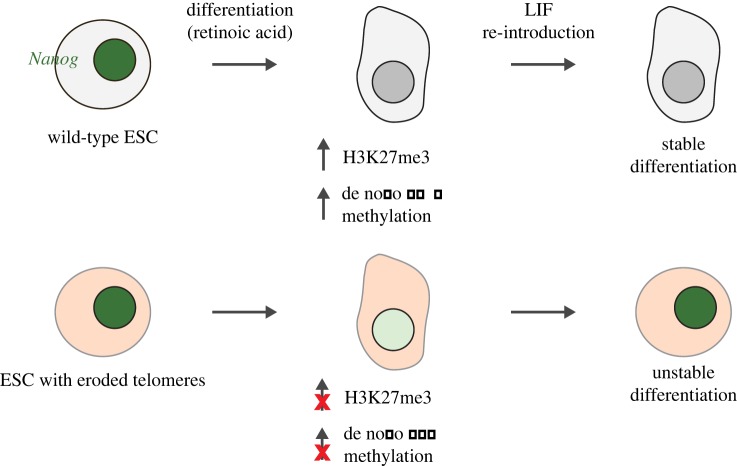
Schematic of the impact of short telomeres on murine embryonic stem cell differentiation. As described in Pucci *et al*. [99], telomere erosion resulted in downregulation of the de novo DNA methyl transferases (Dnmts), and subsequent destabilization of cell differentiation through an inability to suppress the pluripotency factors *Nanog*, *Sox2* and *Oct4/Pou5F1*. Consequently, stem cells were unable to completely and stably respond to differentiation-inducing cues, and could re-acquire pluripotency gene expression and resume cell proliferation after re-addition of the growth factor LIF (leukaemia inhibitory factor).

A direct link between DNA methylation and telomere integrity has been uncovered via the study of stem cell differentiation. Pluripotent murine ESCs can differentiate into cell types of all three germ layers and can efficiently form teratocarcinomas. Pluripotency and self-renewal of ESCs are maintained primarily by the core transcriptional factors Nanog, Oct4 and Sox2 [93], but require the cooperation of other factors and coregulators [94] and an efficient telomere maintenance mechanism [95]. Telomere maintenance is essential for ESC replicative potential [90,96–98] and, during the reprogramming of differentiated cells into stem cells, an increase in telomerase activity leads to telomere elongation and the acquisition of epigenetic marks characteristic of longer telomeres [91,95,98]. Surprisingly though, critically short telomeres appear not only to compromise efficient reprogramming, but also to impair the stability of ESC differentiation [99]. In telomerase-deficient ESCs in fact, an increase in expression of pluripotency factors, including *Nanog* and *Tbx3*, is observed [95,99]. In addition, ESCs and iPS with short telomeres exhibit a reduced expression of the de novo methyltransferases *Dnmt3a* and/or *Dnmt3b* [87,91,95,98–100]. Recently, it was found that *Tert*-deficient ESCs with short telomeres exhibited an unstable response to differentiation-inducing cues such as all-*trans* retinoic acid and, unlike wild-type ESC, could resume proliferation after growth stimulation with the pluripotent cell growth factor LIF [99]. In accord with this apparent reversibility in differentiation status, *Tert*-deficient ESCs cells also exhibited hypomethylation at the *Nanog* and *Oct4*/*Pou5F1* promoters, and the expression of pluripotency factors was elevated. In fact, the total cellular level of DNA methylation was significantly decreased in ESCs with short telomeres. This unstable differentiation phenotype was partially rescued after the elongation of short telomeres by *Tert* reintroduction or by enforced expression of Dnmt3b [99]. These data establish a more wide-ranging impact of critically short telomeres in genome-wide DNA methylation, and suggest that the relationship between short telomeres, ageing, cancer and the methylome might be more intricately linked than originally supposed (figure 2).

Additional recent findings have underscored the link between DNA hypomethylation and unstable differentiation, because knockdown of *de novo* DNA methyltransferases, resulting in genome-wide DNA hypomethylation, prevents consolidation of differentiation programmes and permits reversion to a pluripotent state [101,102]. These data further support the hypothesis that impaired ability to maintain stable differentiation in ESC with short telomeres acts via perturbation of *de novo* DNA methylation, which in turn influences genome-wide chromatin organization and the ability to repress pluripotency factors such as *Nanog*. However, not all epigenetic changes induced by short telomeres lead to DNA hypomethylation. For example, Roberts *et al.* [103] noted that gene silencing of a GFP transgene, concomitant with promoter DNA hypermethylation, was observed in *mTerc^−/−^* mice with short telomeres. Regardless of the precise influence, these data argue that short telomeres may contribute to the already known link between chromatin alterations and ageing [104].

## 5. Alterations in genome-wide expression and cell senescence in yeast

A recent study in yeast also found a marked impact of loss of telomere integrity on gene expression throughout the genome. Using an *S. cerevisiae* strain lacking the telomerase RNA to induce senescence, Platt *et al.* [105] found that telomere attrition resulted in the relocation of the telomere binding protein Rap1 away from telomeres to the promoters of hundreds of genes dispersed throughout the genome, including genes encoding the core histones. This relocation resulted generally in the activation of gene expression, but specifically led to repression in the case of histone genes, concomitant with the onset of senescence. In fact, modulation of Rap1 or core histone levels directly impacted the pace of senescence. The relocation of Rap1 depended on the activity of the DNA damage checkpoint kinase, Mec1, which is activated during senescence concomitant with critical telomere attrition [105]. Other components known to be involved in telomere end-protection, such as the SIR and Ku complexes, are also diverted away from telomeres in response to DNA damage [106], however modulation of SIR activity did not impact the Rap1-dependent alteration of core histone expression in response to imminent senescence [105].

**Figure 3.**
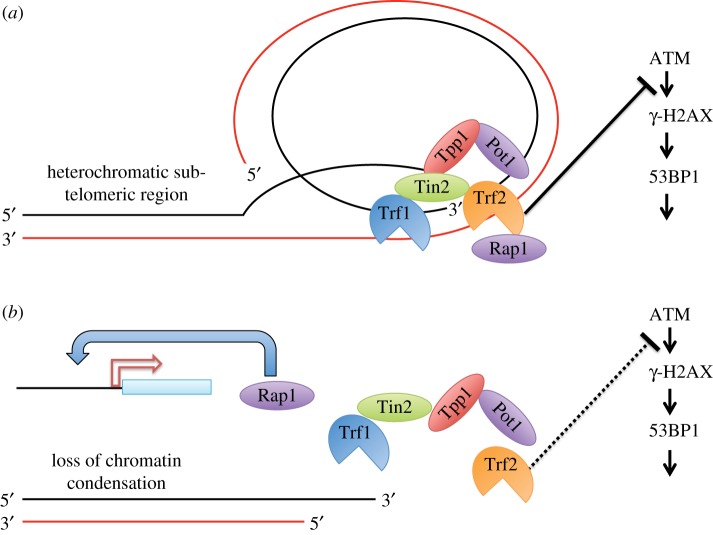
Hypothetical representation of the effects of short telomeres on genome-wide gene expression profiles. Rap1 relocalizes upon cell senescence in yeast [105], and there is also a non-telomeric, transcriptional role of Rap1 in mice [114–116]. Together with other evidence presented in the review, these findings suggest that eroded telomeres might affect genome-wide chromatin rearrangements through a persistent DDR and relocalization of Rap1, or both. In this hypothetical model, at functional telomeres (*a*) the shelterin protein complex (Trf1, Trf2, Rap1, Pot1, Ttp1, Tin2) forms a t-loop and prevents the ATM-dependent activation of DNA DDR mechanisms on the 3′ overhand of telomeres. When telomeres become critically eroded (*b*), changes in the telomeric and sub-telomeric chromatin may lead to a DDR that induces genome-wide alterations in gene expression, e.g. at histones, pluripotency genes or other targets.

Rap1 is an interesting protein with diverse cellular functions. In yeast, it was first discovered based on its role as a Repressor/Activator Protein that acts to regulate gene expression at diverse loci including the mating-type locus [107]. It is also a critical mediator of transcriptional silencing at telomeres and other loci via modulation of chromatin structure [108,109]. Its mammalian counterpart is a component of the telomere protective complex shelterin, and contributes to end-protection and in the suppression of homology-directed repair at telomeres [110,111]. Mice disrupted for *Rap1* (in a genetic background with normal average telomere lengths) reveal an extra-telomeric role of Rap1 in gene expression induced by NF κB signalling [112] and in gene regulation at other subtelomeric and non-telomeric loci [113]. The signalling networks controlled by Rap1 play key roles in metabolic homeostasis, glucose tolerance and adipocyte differentiation [114,115]. Thus, one intriguing possibility raised by these studies is that telomere attrition in mESC with short telomeres might divert Rap1 away from telomeres, and its redistribution to other genomic loci could then lead to changes in DNA methyltransferase expression (figure 3). Indeed, compelling support for this notion was provided by Martinez and colleagues, who found that Rap1 relocalized to other non-telomeric loci in late-generation telomerase-deficient mice [116].

Another non-mutually exclusive possibility, also suggested by Platt *et al.* [105], is that a telomere-induced DDR [117] would trigger changes in the DDR signalling cascade that would lead to a genome-wide change in heterochromatin organization. In support of this notion, a recent study in *S. cerevisiae* showed that a DDR induces global chromatin remodelling and decompaction through loss of histones from chromatin [118]. In human cells, oncogene-induced senescence also induces a DDR, and the concomitant activation of ATM leads to a dramatic relocalization of the histone variant macroH2A1 that, in turn, promotes the senescence-associated secretory phenotype (SASP) and paracrine-induced senescence [119]. Collectively, these results suggest intriguing mechanisms that might underlie the changes in heterochromatin observed in ES and iPS cells with short telomeres. For example, a persistent telomere-induced DDR could gradually lead to loss of histones at widespread chromatin loci, leading to subsequent chromatin decompaction and concomitant changes in genome-wide expression profiles.

## 6. Telomeres could be a missing link between DNA hypomethylation and cell senescence

The above studies establish that telomere shortening can affect DNA methylation elsewhere in the genome, and indeed a reciprocal relationship also exists in murine cells, whereby defects in DNA methylation lead to telomere lengthening [120]. Despite these relationships, it remains to be determined whether critically short telomeres are sufficient to induce the type of methylome changes that have already been described in human aged or cancer cells and tissues. For example, it will be interesting to determine whether some of the footprints associated with the ‘aged methylome’ are directly linked to telomere instability [75].

In human cell culture models, hints at such a link have begun to emerge with recent observations that senescent cells exhibit overall DNA hypomethylation with focal DNA hypermethylation [121], as well as gene silencing and transposable element activation [122]. In one study, senescence-associated DNA hypomethylation occurred at late-replicated genomic regions and was linked to mislocalization of lamin-associated domains that perturbed the expression of the maintenance DNA methyltransferase gene Dnmt1 [121]. In a separate study, reintroduction of *hTERT* into human primary cells led to cellular immortality that was accompanied by a wide-scale reacquisition of DNA methylation and large changes in genome-wide expression profiles [123]. Intriguingly, oncogene-induced bypass of cellular senescence and telomerase-induced bypass result in significantly different DNA methylation and genome-wide expression profiles [123,124]. It is important to note, however, that not all studies found a direct link between telomere status and the epigenome. In one recent study by Lowe and Horvath, the authors found that even telomerase-immortalized cells exhibited evidence of epigenetic ‘ageing’ [125]. With the advent of new methods to measure, modulate and edit the genome/epigenome (such as CRISPR-based gene editing), it should be possible to tease out these complex genetic and epigenetic interrelationships [126,127].

Dysfunctional telomeres also clearly have an impact on cellular phenotypes that arise due to misregulation of genes proximal to short telomeres. As an example, loss of a repressive chromatin state of critically short telomeres in human cells leads to upregulation of DUX4, a primary pathogenic candidate in facioscapulohumeral muscular dystrophy, suggesting that telomere attrition may contribute to disease via perturbation in the ‘telomere position effect’ (TPE: the ability of functional telomeres to impose transcriptional repression of adjacent loci) [128]. Long-range telomere looping has been described in yeast, human and murine cells [129]. Thus, one area of future investigation will be whether the loss of TPE at critically short telomeres could also affect the expression of telomere-distal loci.

It is also worth considering how extracellular secretory mechanisms affected by dysfunctional telomeres may alter global chromatin conformation and gene expression. Mosteiro *et al*. [130] showed that senescence can enhance reprogramming in a mouse model engineered to express the Yamanaka reprogramming factors [131], through the release of inflammatory cytokines, such as interleukin 6, from senescent cells. Interesting, among the murine models used in this study, the authors used mice deficient for the RNA component of telomerase, *Terc*, to induce cellular senescence *in vivo*. Compared to telomerase wild-type mice, *Terc*^−/−^ mice showed a substantial increase in the number of cells undergoing reprogramming following expression of the Yamanaka factors [130]. These data reinforce earlier findings that the alterations in chromatin induced by critically short telomeres clearly do impact pluripotency, both *in vitro* [99] and *in vivo* [130].

## 7. Potential implications for cancer cell differentiation

Because most cancer types often possess a sub-set of critically short chromosome ends within the population [132,133], it is possible that short telomeres would contribute to greater plasticity in cancer cell fate, and may promote resistance to normal differentiation-inducing cues. Drugs or agents that promote differentiation, such as all-*trans* retinoic acid, have become a standard cancer treatment for many different types of cancer, including blood (leukaemia), breast, prostate, and glioblastoma [86]. Despite the success of this approach, a fraction of cancers relapse due to the emergence of proliferative cells that are differentiation-resistant, for example, in acute promyelocytic leukaemia [86]. Similarly, prostate cancer cells, which possess very short average telomere lengths, exhibit an alteration in gene expression profiles and morphology not unlike those of their lineage-committed, differentiated epithelial cell precursors [134]. These data support the hypothesis that the differentiation resistance of cancers may be influenced directly by telomere integrity. In one prostate cancer cell line, enforced telomere elongation via ectopic expression of TERT promoted the acquisition of expression patterns reminiscent of more differentiated lineages when injected into nude mice *in vivo*. This effect depended on the catalytic competence of TERT and was not observed when catalytically inactive TERT was introduced into the same cell line [134].

Cell-based studies have their merits, but it is whole animal genetic models that will permit a direct test of the hypothesis that critically short telomeres induce DNA methylation changes that impact cell and tissue physiology. An age-associated increase in heterochromatic modifications has been noted in murine and primate models, but the role of telomeres in this process is still unknown [135]. The physiological consequence of DNA methylation changes induced by short telomeres could be tested in telomerase knockout mice, in which telomere loss culminates in wide-scale declines in stem cell and tissue function that lead to sterility and early mortality [136]. These changes are reversible via genetic or chemically inducible rescue of endogenous TERT expression, which results in re-extension of telomeres, reduced DNA damage signalling and associated cellular checkpoint responses and the amelioration of degenerative phenotypes in several tissues including brain, testes, spleen and intestines [137–139]. However, reintroduction of *Tert* can also promote aggressive cancer development and bone metastasis in *Tert* knockout mice [140]. It will be interesting to determine whether this rescue of TERT function is also accompanied by reprogramming of the epigenome in normal and pre-neoplastic cell types, similar to the changes observed during replicative senescence in human cells, or whether there is a methylome footprint associated with age or cancer that is conserved between mice and humans [75]. For example, although critically eroded telomeres do accelerate haematopoietic stem cell (HSC) exhaustion *in vivo* during serial bone marrow transplantation [141], in ageing human HSCs, large-scale changes in the DNA methylation landscape occurred in an age-dependent manner, but these changes did not correlate with detectable alterations in telomere length (although it should be noted that the PCR-based technique used to measure telomere length does not detect critically short telomeres) [142]. Nonetheless, it would be naïve to assume that all age-associated changes in the genome are linked to telomere status.

In summary, we have outlined a recent sampling of the unusual consequences of excessive telomere erosion or elongation that may affect cell function. These discoveries underscore the importance of continual re-examination of what we may have previously considered to be well-examined or ‘answered’ questions in biology. ‘Look and you will find it—what is unsought will go undetected’ (Sophocles). With the advent of sophisticated technologies to assess genome-wide changes in histone post-translational status, DNA methylation and RNA expression, who knows where the contribution of telomeres will end?

## Data accessibility

This article has no additional data.
